# Lobbying Expenditures and Campaign Contributions by the Pharmaceutical and Health Product Industry in the United States, 1999-2018

**DOI:** 10.1001/jamainternmed.2020.0146

**Published:** 2020-03-03

**Authors:** Olivier J. Wouters

**Affiliations:** 1 Department of Health Policy, London School of Economics and Political Science, London, United Kingdom

## Abstract

**Importance:**

Government efforts to lower drug costs and other legislative and regulatory initiatives may be counteracted by campaign donors and lobbyists in the pharmaceutical and health product industry.

**Objective:**

To review how much money the pharmaceutical and health product industry spent on campaign contributions and lobbying in the US from 1999 to 2018 at the federal and state levels.

**Design and Setting:**

Analysis of federal-level and state-level data obtained from the Center for Responsive Politics and the National Institute on Money in Politics, respectively. These nonprofit, nonpartisan organizations track federal and state campaign contributions and lobbying expenditures by individuals and groups.

**Exposures:**

Lobbying expenditures and contributions to political campaigns.

**Main Outcomes and Measures:**

Total spending, inflation adjusted to 2018 dollars using the US Consumer Price Index, on lobbying and campaign contributions by year, source, and state.

**Results:**

From 1999 to 2018, the pharmaceutical and health product industry recorded $4.7 billion—an average of $233 million per year—in lobbying expenditures at the federal level, more than any other industry. Of the spending, the trade group Pharmaceutical Research and Manufacturers of America accounted for $422 million (9.0%), and the other 19 top companies and organizations in this industry accounted for $2.2 billion (46.8%). The industry spent $414 million on contributions to candidates in presidential and congressional elections, national party committees, and outside spending groups. Of this amount, $22 million went to presidential candidates and $214 million went to congressional candidates. Of the 20 senators and 20 representatives who received the most contributions, 39 belonged to committees with jurisdiction over health-related legislative matters, 24 of them in senior positions. The industry contributed $877 million to state candidates and committees, of which $399 million (45.5%) went to recipients in California and $287 million (32.7%) went to recipients in 9 other states. In years in which key state referenda on reforms in drug pricing and regulation were being voted on, there were large spikes in contributions to groups that opposed or supported the reforms.

**Conclusions and Relevance:**

From 1999 to 2018, the pharmaceutical and health product industry spent large sums of money on lobbying and campaign contributions to influence legislative and election outcomes. These findings can inform discussions about how to temper the influence of industry on US health policy.

## Key Points

**Question:**

How much money did the pharmaceutical and health product industry spend on lobbying and campaign contributions in the US from 1999 to 2018?

**Findings:**

This observational study, which analyzed publicly available data on campaign contributions and lobbying in the US from 1999 to 2018, found that the pharmaceutical and health product industry spent $4.7 billion, an average of $233 million per year, on lobbying the US federal government; $414 million on contributions to presidential and congressional electoral candidates, national party committees, and outside spending groups; and $877 million on contributions to state candidates and committees. Contributions were targeted at senior legislators in Congress involved in drafting health care laws and state committees that opposed or supported key referenda on drug pricing and regulation.

**Meaning:**

An understanding of the large sums of money the pharmaceutical and health product industry spends on lobbying and campaign contributions can inform discussions about how to temper the influence of industry on US health policy.

## Teaser

This observational study uses publicly available data to analyze how much the pharmaceutical and health product industry spent on campaign contributions and lobbying in the US from 1999 to 2018.

## Introduction

In 2018, the US spent an estimated $3.6 trillion, or 17.6% of its $20.5-trillion gross domestic product, on health care, including $345 billion on prescription drugs sold in retail pharmacies.^1^ Adjusted for inflation, per-person spending on prescription drugs sold in US retail pharmacies increased from $520 in 1999 to $1025 in 2017.^2^ Although both Democrats and Republicans consider lowering prescription drug prices a priority,^3^ lobbyists and campaign donors in the pharmaceutical industry may counteract efforts by federal and state governments to decrease these costs.

In the US, citizens and organizations, including corporations, have the right to petition politicians and elected officials to try to influence policy decisions.^4^ Citizens and organizations may do so individually or collectively through interest groups. They can exert their influence through lobbying (ie, contacts by paid lobbyists with officials or their staff to disseminate information about regulatory or legislative matters).^4,5^ Apart from lobbying, individuals and organizations may contribute money to political campaigns to support their preferred candidates and improve their access to successful candidates.^6,7,8^ Campaign contributions and lobbying expenditures differ and are subject to different regulations.^8,9^

There is evidence that campaign contributions and lobbying expenditures may influence election and legislative outcomes.^10,11,12,13,14,15^ However, few studies have analyzed such spending by the pharmaceutical and health product industry, and most of the research is from 2009 or earlier.^16,17,18,19,20,21,22^ Prior research has primarily focused on lobbying and campaign contributions by the health care sector as a whole in individual years at the federal level. Trends over time have received less attention, as have contributions to candidates and committees in state elections, where money may be used to influence the outcomes of referenda on measures aimed at lowering drug costs.

This study analyzed lobbying expenditures and patterns of election contributions by the pharmaceutical and health product industry at the federal and state levels from 1999 to 2018.

## Methods

Federal-level and state-level data were obtained from the Center for Responsive Politics^23^ and the National Institute on Money in Politics,^24^ respectively. These nonprofit, nonpartisan organizations track federal and state campaign contributions and lobbying expenditures by individuals and groups.

Both organizations categorized all contributions and expenditures by sector and industry within each sector; the categories were modeled on the federal government’s standard industrial classification system. The pharmaceutical and health product industry includes manufacturers of pharmaceutical and biological products, diagnostic tests, medical devices and equipment, and nutritional and dietary supplements as well as pharmacy benefit managers.

On September 30, 2019, the databases of the 2 organizations were searched for campaign contributions and lobbying expenditures by individuals and groups in the pharmaceutical and health product industry from January 1, 1999, to December 31, 2018. State-level data on campaign contributions from January 1, 1999, to December 31, 2002, were incomplete for some states owing to lack of reporting. As no data were collected from human participants and the data were publicly available, the study was exempt from institutional review board approval at the London School of Economics and Political Science.

### Federal-Level Data

Data on lobbying expenditures were based on disclosure reports filed with the Senate Office of Public Records. Lobbying firms are required to provide the office with quarterly estimates of lobbying incomes (rounded to the nearest $10 000) from clients who spent $3000 or more in a given quarter. Organizations that hire lobbyists as direct employees are required to report lobbying-related expenditures to the nearest $10 000 if outlays were $12 500 or more in a given quarter.^23^

The data on election campaign contributions were based on disclosure reports filed with the Federal Election Commission. From these reports, the Center for Responsive Politics extracted all records of (1) cash contributions of $200 or more to federal candidates and national party committees from individual donors and political action committees; (2) soft money contributions from individuals, corporations, labor unions, and ideological groups to national party committees; and (3) donations to outside spending groups, which operate independently of and not in coordination with candidates’ committees and can spend money on communications with the public. Soft money contributions from individuals, corporations, labor unions, and ideological groups to national party committees are donations that cannot be used to directly support the election bids of federal candidates but rather fund other initiatives, such as voter registration drives.^16^

In 2002, the Bipartisan Campaign Reform Act^25^ banned soft money contributions but allowed donations to state and local political parties for use in activities related to voter registration and participation in federal elections, known as Levin funds. Congress acted in response to concerns that soft money contributions, which were not subject to federal limits on campaign contributions, were being misappropriated.^16^ The 2010 Supreme Court case of *Citizens United v Federal Election Commission*^26^ (and related court decisions) legalized contributions by corporations and unions to new types of outside spending groups, including so-called super political action committees. Prior to the ruling, no donations to these groups were recorded; the ban on soft money contributions remained in effect.

For each of the 20 senators and 20 representatives who received the most contributions from the pharmaceutical and health product industry from 1999 to 2018, records from the US Government Publishing Office^27^ were searched to determine whether these members served at any point during this period on a committee with jurisdiction over health-related legislative matters, a health-related subcommittee of one of these committees, or both.^27^ It was also noted if a member served as chair, vice chair, or ranking member of any of these committees or subcommittees or held a party leadership position (Speaker of the House, majority leader, minority leader, majority whip, or minority whip in the House; president pro tempore, majority leader, minority leader, majority whip, or minority whip in the Senate). Some committees and subcommittees changed names over the study period.

For the House of Representatives, the committees were Energy and Commerce; Ways and Means; Oversight and Reform; Budget; Education and Labor; Appropriations; and Veterans’ Affairs. For the Senate, the committees were Finance; Aging (Special Committee); Budget; Health, Education, Labor, and Pensions; Appropriations; and Veterans’ Affairs.

The subcommittees included in the House were (1) Health (Energy and Commerce); (2) Health (Ways and Means); (3) Health Care, Benefits, and Administrative Rules (Oversight and Reform); (4) Health, Employment, Labor, and Pensions (Education and Labor); (5) Agriculture, Rural Development, Food and Drug Administration, and Related Agencies (Appropriations); (6) Labor, Health and Human Services, Education, and Related Agencies (Appropriations); (7) Department of Veterans’ Affairs (Appropriations); and (8) Health (Veterans’ Affairs). In the Senate, the subcommittees were (1) Health Care (Finance); (2) Primary Health and Retirement Security (Health, Education, Labor, and Pensions); (3) Agriculture, Rural Development, Food and Drug Administration, and Related Agencies (Appropriations); (4) Labor, Health and Human Services, Education, and Related Agencies (Appropriations); and (5) Military Construction, Veterans’ Affairs, and Related Agencies (Appropriations).

### State-Level Data

Data on lobbying expenditures at the state level were unavailable for most states and thus were excluded from the analysis. However, the National Institute on Money in Politics^24^ collects data from all 50 states on campaign contributions from individuals and organizations in the pharmaceutical and health product industry to the following state-level candidates and committees: (1) gubernatorial or other statewide candidates; (2) house, assembly, or senate candidates; (3) supreme court candidates; (4) political party committees; and (5) ballot measure committees. Ballot measure committees raise funds to oppose or support ballot measures, which are proposals that are voted on by the electorate to pass or repeal state laws or amendments to the state constitution.^28^ The National Institute on Money in Politics^24^ acquires the data from various state regulatory offices.

### Data Analysis

Descriptive statistics were used to report total campaign contributions and lobbying expenditures by the pharmaceutical and health product industry, with results broken down by year, source, recipient, political party, and state. For comparison, aggregated data on federal lobbying expenditures by the top 10 industries and organizations were also collected, as were data on expenditures by the 4 industries in the health sector in addition to the pharmaceutical and health product industry (ie, hospitals and nursing homes, health professionals, health services and health maintenance organizations, and miscellaneous health organizations). Because health insurance companies are grouped with life, property, and car insurance firms, they were excluded from the health sector for this analysis. Federal campaign contributions were recorded over 2-year cycles, reflecting the timing of congressional elections.

All dollar figures were inflation adjusted to 2018 dollars using the US Consumer Price Index. Stata version 15 (StataCorp) and Excel 2016 (Microsoft) were used for all analyses.

## Results

### Federal-Level Lobbying Expenditures

From 1999 to 2018, across all industries, a total of $64.3 billion was spent lobbying Congress and federal agencies in the US. During this time, the pharmaceutical and health product industry recorded the highest spending of all industries ($4.7 billion [7.3%]), followed by the insurance industry ($3.2 billion [5.0%]), the electric utilities industry ($2.8 billion [4.4%]), and the electronics manufacturing and equipment industry ($2.6 billion [4.0%]). Within the health sector, total lobbying expenditures were $9.7 billion. Expenditures in addition to those by the pharmaceutical and health product industry were recorded by hospitals and nursing homes ($1.9 billion), health care professionals ($1.7 billion), health services and health maintenance organizations ($1.3 billion), and miscellaneous health organizations ($139 million).

**Figure 1.**
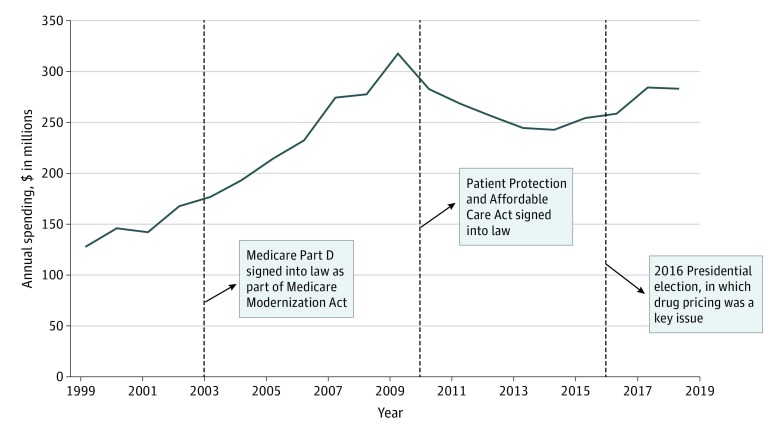
Federal-Level Lobbying Expenditures by the Pharmaceutical and Health Product Industry, 1999-2018 Dashed lines indicate key events that affected the pharmaceutical and health product industry. Data from the Center for Responsive Politics.^23^ Amounts were inflation adjusted to 2018 dollars using the US Consumer Price Index.

Pharmaceutical and health product industry spending on federal lobbying averaged $233 million per year. From 1999 to 2009, annual spending increased, before decreasing briefly and increasing again (Figure 1). Expenditures peaked at $318 million in 2009, the year before the Patient Protection and Affordable Care Act was signed into law.

Over the 20-year study period, 1375 organizations in the pharmaceutical and health product industry reported lobbying expenditures. Seventeen of the 20 highest spending organizations were manufacturers of biological or pharmaceutical products or their trade associations (Table 1). The other 3 organizations were the Advanced Medical Technology Association (a trade association for medical device companies), Medtronic (a medical device company), and the Seniors Coalition (an interest group that does not disclose its donors and lobbies for limited government intervention in drug and health care markets). The top spender, the trade group Pharmaceutical Research and Manufacturers of America (PhRMA), accounted for $422 million (9.0%) of the $4.7 billion, and the other 19 top spenders accounted for $2.2 billion (46.8%).

**Table 1.** Top 20 Lobbying Spenders and Campaign Contributors in the Pharmaceutical and Health Product Industry at the Federal Level, 1999-2018^a^

Rank	Organization^b^	Expenditures, $ in millions
**Lobbying spenders**		
1	Pharmaceutical Research and Manufacturers of America	422.3
2	Pfizer	219.2
3	Amgen	192.7
4	Eli Lilly and Company	166.2
5	Biotechnology Innovation Organization (BIO)^c^	153.4
6	Merck	143.0
7	Roche Holdings^c^	135.9
8	Novartis	130.2
9	Johnson & Johnson	129.9
10	Sanofi^c^	116.7
11	Bayer	111.0
12	GlaxoSmithKline	110.8
13	Bristol-Myers Squibb	101.6
14	Abbott Laboratories	96.6
15	Advanced Medical Technology Association	79.4
16	Seniors Coalition	65.3
17	Medtronic	63.8
18	Baxter International	58.4
19	AstraZeneca	54.6
20	Teva Pharmaceutical Industries	53.3
Total		2604.3
**Campaign contributors**^**d**^		
1	Pfizer	23.2
2	Amgen	14.7
3	Eli Lilly and Company	13.3
4	GlaxoSmithKline	12.6
5	SlimFast Foods	11.3
6	Johnson & Johnson	11.2
7	D.E. Shaw Research	11.0
8	Merck	10.6
9	Abbott Laboratories	10.0
10	Bristol-Myers Squibb	7.7
11	Exoxemis	6.9
12	McKesson	6.8
13	Ischemix	5.7
14	Pharmaceutical Research and Manufacturers of America	5.6
15	AstraZeneca	5.4
16	Pharmaceutical Product Development	5.2
17	Schering-Plough	5.1
18	AmerisourceBergen	4.9
19	Sanofi^c^	4.3
20	Novartis	4.0
Total		179.5

^a^ Data from the Center for Responsive Politics.^23^ Amounts were inflation adjusted to 2018 dollars using the US Consumer Price Index.

^b^ Expenditures by subsidiary organizations were attributed to the parent organizations. Amounts included contributions from organizations’ political action committees and from individuals. Companies that merged or were acquired were treated as separate entities prior to the transaction.

^c^ BIO changed its name from Biotechnology Industry Organization to Biotechnology Innovation Organization in 2016; the figure for BIO included expenditures under both names. The figure for Roche Holdings included expenditures by Roche Group. Sanofi changed its name from Sanofi-Aventis to Sanofi in 2011; the figures for Sanofi included expenditures under both names.

^d^ Amounts included contributions to candidates, party committees, and outside spending groups. These figures included contributions from organizations’ political action committees and from individual members, employees, or owners of companies or organizations in an industry or from their immediate family members.

Across all industries, only 5 organizations reported more spending than PhRMA: the US Chamber of Commerce ($1.7 billion), the National Association of Realtors ($602 million), the American Medical Association ($462 million), the American Hospital Association ($426 million), and General Electric ($423 million). Following PhRMA, the seventh and eighth ranked spenders were the Blue Cross Blue Shield Association ($391 million) and AARP (formerly American Association of Retired Persons) ($334 million). Thus, 5 of 8 organizations with the largest lobbying expenditures were health care related.

### Federal-Level Campaign Contributions

**Figure 2.**
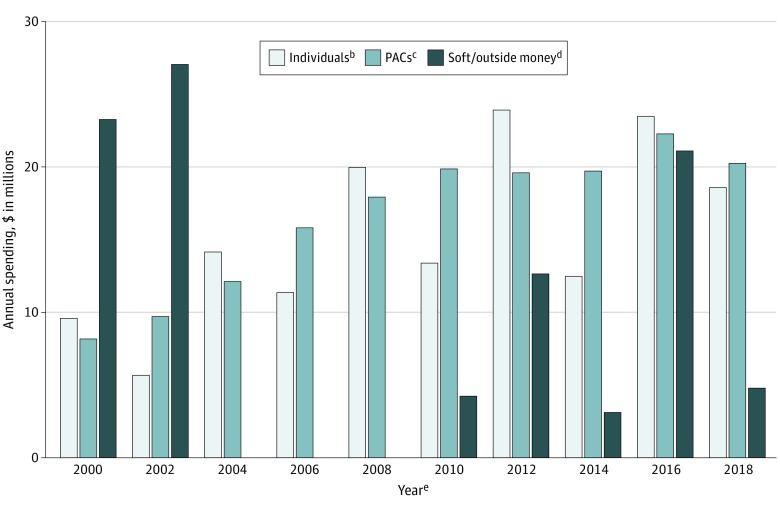
Campaign Contributions by the Pharmaceutical and Health Product Industry to Federal (Presidential and Congressional) Elections by Source, 1999-2018^a^ PAC indicates political action committee. ^a^Data from the Center for Responsive Politics.^23^ Amounts were inflation adjusted to 2018 dollars using the US Consumer Price Index. ^b^Contributions from individual members, employees, or owners of companies or organizations in an industry or from their immediate family members; there are limits on individual contributions to candidates and national party committees during elections. ^c^PACs pool campaign contributions from members of corporations, labor unions, and ideological groups and disburse the funds to political candidates and national party committees; there are limits on PAC contributions to candidates and national party committees during elections. ^d^Soft money contributions (banned as of November 6, 2002) and donations to outside spending groups and Levin funds. Outside spending groups, which include so-called super PACs, operate independently of and not in coordination with candidates’ committees; spending by outside groups is largely unregulated and unlimited. ^e^Each year corresponds to a 2-year election cycle; eg, 2000 refers to January 1, 1999, through December 31, 2000. Presidential elections occurred in 2000, 2004, 2008, 2012, and 2016.

From 1999 to 2018, the pharmaceutical and health product industry contributed $414 million to federal (presidential and congressional) candidates, national party committees, and outside spending groups (Figure 2). This included $152 million in contributions from individuals affiliated with the health care industry, $165 million from political action committees, and $96 million in soft money contributions and donations to outside spending groups.

Excluding contributions to outside spending groups, the industry donated $367 million to party candidates and committees ($216 million [58.9%] to Republicans; $151 million [41.1%] to Democrats), with more money going to Republicans than to Democrats in all but 2 election cycles (2008 and 2010). The 2000 and 2002 election cycles, 2 of 5 cycles with the highest spending levels, coincided with congressional debates on the introduction of Medicare Part D (a prescription drug benefit program for seniors) and the 2000 presidential election. The 2 cycles with the highest spending (2012 and 2016) were presidential election years. The 2018 election cycle had the fifth highest spending.

Of the top 20 campaign contributors (Table 1), 15 were manufacturers of biological or pharmaceutical products, and 1 was the trade group PhRMA. The other 4 were AmerisourceBergen (a drug wholesale company), D.E. Shaw Research (a biochemistry research company), Pharmaceutical Product Development (a contract research organization), and SlimFast Foods (a producer of nutritional and dietary supplements). Five pharmaceutical companies were among the top 10 spenders for both campaign contributions and lobbying: Amgen, Eli Lilly and Company, Johnson & Johnson, Merck, and Pfizer.

Contributions to presidential candidates totaled $22 million. The eTable in the Supplement lists the 20 presidential candidates who received the most contributions from individuals and political action committees in the pharmaceutical and health product industry. Of the $19.3 million contributed to these candidates, the top recipient was Barack Obama ($5.5 million), followed by Hillary Clinton ($3.7 million), Mitt Romney ($3.0 million), and George W. Bush ($2.4 million). The next 16 candidates combined received $4.7 million.

Contributions to congressional candidates totaled $214 million. Table 2 shows the top 20 recipients, in each chamber of Congress, of contributions from individuals and political action committees in the pharmaceutical and health product industry. These 40 legislators jointly received $45 million (21.0%) of all contributions to congressional candidates; 39 were members of committees with jurisdiction over health-related legislative matters, and 24 held senior positions in these committees. Of the 20 members of the House, 17 served on the Energy and Commerce Committee or the Ways and Means Committee. Of the 20 senators, 13 served on the Finance Committee.

**Table 2.** Top Recipients of Campaign Contributions From the Pharmaceutical and Health Product Industry in Congressional Elections, 1999-2018^a^

Rank	Candidate (party, state)	Contributions received, $ in millions^b^	Active years	Member of health-related committee^c^	Senior member of health-related committee^d^	Party leader^e^
**House elections**						
1	Eshoo, Anna (D, California)	1.8	1993-present	Yes^f^	No^g^	No
2	Upton, Fred (R, Michigan)	1.6	1987-present	Yes^f^	Yes	No
3	Pallone, Frank (D, New Jersey)	1.5	1988-present	Yes^f^	Yes	No
4	McCarthy, Kevin (R, California)	1.4	2007-present	No	No	Yes
5	Paulsen, Erik (R, Minnesota)	1.3	2009-2019	Yes^f^	No	No
6	Ryan, Paul (R, Wisconsin)	1.3	1999-2019	Several^f^	Yes	Yes
7	Ferguson, Mike (R, New Jersey)	1.3	2001-2009	Yes^f^	Yes	No
8	Boehner, John (R, Ohio)	1.3	1991-2015	Yes	Yes	Yes
9	Walden, Greg (R, Oregon)	1.2	1999-present	Several	Yes	No
10	Shimkus, John (R, Illinois)	1.2	1997-present	Yes^f^	No	No
11	Hoyer, Steny (D, Maryland)	1.1	1981-present	Yes^f^	No	Yes
12	Barton, Joe (R, Texas)	1.0	1985-2019	Yes^f^	Yes	No
13	Burgess, Michael (R, Texas)	1.0	2003-present	Yes^f^	Yes	No
14	Tiberi, Pat (R, Ohio)	1.0	2001-2018	Several^f^	Yes	No
15	Dingell, John (D, Michigan)	1.0	1955-2015	Yes^f^	Yes	No
16	Lance, Leonard (R, New Jersey)	1.0	2009-2019	Yes^f^	No	No
17	Camp, Dave (R, Michigan)	1.0	1991-2015	Yes^f^	Yes	No
18	Johnson, Nancy (R, Connecticut)	0.9	1983-2007	Yes^f^	Yes	No
19	Cantor, Eric (R, Virginia)	0.9	2001-2014	Yes	No	Yes
20	Kind, Ron (D, Wisconsin)	0.9	1997-present	Several^f^	No	No
Total		23.7	NA	19	12	5
**Senate elections**						
1	Hatch, Orrin (R, Utah)	2.8	1977-2019	Several^f^	Yes	Yes
2	Burr, Richard (R, North Carolina)	1.6	2005-present	Several^f^	Yes	No
3	McConnell, Mitch (R, Kentucky)	1.4	1985-present	Yes^f^	No	Yes
4	Casey, Bob (D, Pennsylvania)	1.3	2007-present	Several^f^	Yes	No
5	Clinton, Hillary (D, New York)	1.2	2001-2009	Several^f^	No	No
6	Murray, Patty (D, Washington)	1.1	1993-present	Several^f^	Yes	No
7	Baucus, Max (D, Montana)	1.1	1978-2014	Yes^f^	Yes	No
8	Schumer, Charles (D, New York)	1.0	1999-present	Yes^f^	No	Yes
9	Portman, Rob (R, Ohio)	1.0	2011-present	Several^f^	No	No
10	Specter, Arlen (R, Pennsylvania)	1.0	1981-2011	Several^f^	Yes	No
11	Grassley, Chuck (R, Iowa)	0.9	1981-present	Several^f^	Yes	Yes
12	Menendez, Robert (D, New Jersey)	0.9	2006-present	Several^f^	No	No
13	Cornyn, John (R, Texas)	0.9	2002-present	Several^f^	No	Yes
14	Santorum, Rick (R, Pennsylvania)	0.8	1995-2007	Several^f^	No	No
15	Wyden, Ron (D, Oregon)	0.8	1996-present	Several^f^	Yes	No
16	Harkin, Tom (D, Iowa)	0.8	1985-2015	Several^f^	Yes	No
17	Alexander, Lamar (R, Tennessee)	0.8	2003-present	Several^f^	Yes	No
18	Toomey, Pat (R, Pennsylvania)	0.8	2011-present	Several^f^	Yes	No
19	Isakson, Johnny (R, Georgia)	0.7	2005-present	Several^f^	Yes	No
20	Reid, Harry (D, Nevada)	0.7	1987-2017	Several^f^	No	Yes
Total		21.4	NA	20	12	6

Abbreviations: D, Democrat; NA, not applicable; R, Republican.

^a^ The table reflects congressional positions held at any point during the study period (January 1, 1999, through December 31, 2018). Some committees and subcommittees changed names over the study period. Data on committee memberships and Congressional positions were obtained from the US Government Publishing Office.^27^

^b^ Data obtained from the Center for Responsive Politics.^23^ Monetary amounts were inflation adjusted to 2018 dollars using the US Consumer Price Index.

^c^ See the Federal-Level Data section in the Methods section for a listing of committees.

^d^ The member was the chair, vice chair, or ranking member of at least 1 health-related committee or health-related subcommittee. See the Federal-Level Data section in the Methods section for a listing of committees and subcommittees.

^e^ The member held at least 1 party leadership position in Congress (Speaker of the House, majority leader, minority leader, majority whip, or minority whip in the House; president pro tempore, majority leader, minority leader, majority whip, or minority whip in the Senate).

^f^ The member served on at least 1 of the selected committees and at least 1 health-related subcommittee.

^g^ Anna Eshoo is currently chair of the Health Subcommittee in the House Energy and Commerce Committee (2019-present).

### State-Level Campaign Contributions

From 1999 to 2018, the pharmaceutical and health product industry contributed $877 million to state-level candidates and committees in 50 states and the District of Columbia, of which $661 million (75.4%) went to ballot measure committees. The remainder was contributed to house of representatives, assembly, and senate candidates ($99 million), state party committees ($72 million), gubernatorial and other statewide candidates ($44 million), and supreme court candidates ($1 million).

Over this period, total contributions exceeded $50 million in 2 states, California ($399 million) and Ohio ($74 million), and were between $20 million and $50 million in 6 states—Missouri ($43 million), New York ($33 million), Oregon ($27 million), Florida ($26 million), Illinois ($23 million), and Texas ($22 million). Contributions totaled $10 million to less than $20 million in 7 states, $5 million to less than $10 million in 7 states, $1 million to less than $5 million in 20 states, and less than $1 million in 8 states and the District of Columbia. Candidates and committees in California received 45.5% ($399 million) of all contributions, compared with 32.7% ($287 million) for recipients in the other 9 states with the most contributions.

**Figure 3.**
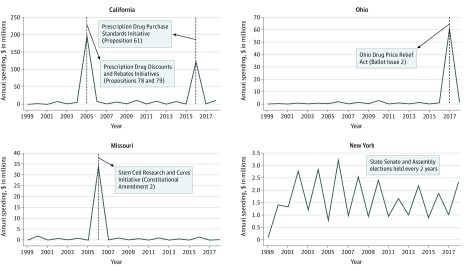
State-Level Contributions by the Pharmaceutical and Health Product Industry to Candidates, Party Committees, and Ballot Measure Committees in Top 4 States, 1999-2018 Dashed lines show years with key ballot measures that affected the industry. From 1999 to 2018, the pharmaceutical and health product industry contributed $399 million in California, $74 million in Ohio, $43 million in Missouri, and $33 million in New York. Contributions from 1999 to 2002 may be underestimated because of incomplete data. Data from the National Institute on Money in Politics.^24^ Amounts were inflation adjusted to 2018 dollars using the US Consumer Price Index.

Figure 3 shows trends in contributions in the 4 states that received the most money. Of the $399 million in contributions in California, $197 million (49.4%) and $123 million (30.8%) were spent in 2005 and 2016, respectively; in these 2 years, there were 3 ballot measures intended to reduce drug costs, all of which were rejected by voters.^29,30,31,32^ In 2005, 1 of 2 defeated ballot measures was Proposition 78, which PhRMA and pharmaceutical companies supported. In the other years, contributions in California followed cyclical patterns, reflecting the timing of legislative elections. Of the $74 million donated in Ohio, $61 million (82.4%) was spent in 2017, the year of a ballot measure aimed at lowering prescription drug costs, which was voted down.^33^ Of the $43 million donated in Missouri, $34 million (79.1%) was spent in 2006, the year of a ballot measure on the legality of stem cell research, which was passed.^34^ Contributions in New York followed a cyclical pattern in line with the timing of state senate and assembly elections. Trends in the other 46 states and the District of Columbia generally followed the pattern observed in New York, with a few exceptions.

## Discussion

From 1999 to 2018, the pharmaceutical and health product industry spent large sums of money on lobbying and campaign contributions. More than twice as much money was spent on elections at the state level than at the federal level. Federal campaign contributions were targeted at senior legislators serving on congressional committees that draft health care bills and at presidential candidates from both major political parties. At the state level, the industry focused its efforts on opposing major drug cost-containment measures by contributing to ballot measure committees in key states. Three cost-containment ballot measures in California and 1 in Ohio were all defeated.^30,31,32,33^ In 2005, PhRMA and pharmaceutical companies supported 1 of the ballot measures, Proposition 78 in California; the proposition, which voters rejected, would have allowed pharmaceutical companies to voluntarily provide discounts on drugs sold to individuals with an income below a threshold.^30^

When considering legislative and policy initiatives, Congress and the executive branch benefit from fully considering the interests of all parties in society, not just those who seek to improve their access to officials through campaign contributions and lobbying expenditures. In the health sector, several organizations, notably PhRMA, the American Medical Association, the American Hospital Association, and the Blue Cross Blue Shield Association, accounted for a disproportionate share of spending on lobbying over the study period. PhRMA and the American Medical Association have historically lobbied together against government interventions in drug markets.^35,36,37,38^ For example, although both groups supported the Affordable Care Act, they did so only after receiving commitments from the Obama administration and former Senator Max Baucus (D, Montana), then chair of the Senate Finance Committee, that parallel import of lower-cost medicines from Canada would not be permitted, Medicare would not be allowed to negotiate drug prices, and Medicare payments to physicians would not be reduced.^35^ With the exception of a few influential consumer groups that have lobbied Congress to lower drug prices—such as AARP—groups representing consumers spent far less on lobbying than industry trade groups and companies.

The $4.7 billion spent by the pharmaceutical and health product industry on lobbying and the $1.3 billion spent on campaign contributions from 1999 to 2018 was only about 0.1% of the estimated $5.5 trillion (in 2018 dollars) spent on prescription drugs in the US over the same period.^39^ As a percentage of their revenues, well-resourced drug industry groups had to spend relatively little in their efforts to influence political and legislative outcomes. In contrast, many organizations advocating for the interests of patients and consumers have more limited financial resources.

Legislative and regulatory changes might address some of the disparities highlighted by this analysis. Such changes might include restrictions on donations by individuals and organizations to ballot measure committees at the state level. At present, in many states, including California^40^ and Ohio,^41^ these committees are not subject to contribution limits. Transparency about financial associations might also be increased, particularly for congressional leaders and members of committees that draft legislation affecting the pharmaceutical and health product industry and other aspects of health care. For example, Congress could mandate that chairs and ranking members of health-related committees publish online, in a readily accessible manner and in a format understandable to the electorate, records of scheduled meetings with lobbyists from relevant industries, as is required by the European Parliament.^42^

### Limitations

This analysis had limitations. First, it was not possible to verify the completeness of the data. However, the nonpartisan organizations from which the data were obtained conduct extensive validation and triangulation of sources to ensure accuracy.^23,24^ Even so, the data did not capture all lobbying activities because some expenditures fell outside of the disclosure requirements (eg, small outlays and certain indirect expenses, such as investments in buildings and infrastructure).

Second, there were inconsistencies among companies in the reporting of lobbying expenditures, which made it difficult to ensure the comparability of figures. Organizations in the pharmaceutical and health product industry report federal lobbying incomes or expenditures to the Senate Office of Public Records through 1 of 3 filing methods.^23^ The first 2 methods adhere to the definition of lobbying in the Internal Revenue Code (1 method for for-profit groups and 1 for nonprofit groups),^43,44^ whereas the third method follows the definition in the Lobbying Disclosure Act of 1995.^45^ The 2 filing methods based on the Internal Revenue Code definition require filers to disclose state and grassroots lobbying costs alongside federal lobbying costs, whereas the other method does not. Moreover, the Lobbying Disclosure Act of 1995 definition covers a larger number of public officials than the definition in the Internal Revenue Code.

Third, in any given year, the Center for Responsive Politics^23^ and the National Institute on Money in Politics^24^ were unable to categorize approximately 30% and 15%, respectively, of dollars spent on campaign contributions from individuals by industry because of lack of information. Thus, contributions at both state and federal levels may have been underestimated for the pharmaceutical and health product industry.^24,46^ The 2 organizations categorize contributions from individual donors based on self-reported employment information.

Fourth, at a state level, data on lobbying expenditures were excluded from this analysis owing to unavailability, and data on campaign contributions from 1999 to 2002 were likely underestimated because of incomplete reporting. Also, state-level data on campaign contributions excluded independent spending (ie, money spent on communications with the public by individuals or organizations that operated independently of and not in coordination with candidates’ committees). This included spending on direct advocacy communications (ie, “a communication, such as a website, newspaper, TV or direct mail advertisement that expressly advocates the election or defeat of a clearly identified candidate”^47^), electioneering communications (ie, “any broadcast, cable or satellite communication that refers to a clearly identified…candidate, is publicly distributed within 30 days of a primary or 60 days of a general election and is targeted to the relevant electorate”^48^), and internal communications targeted to members of a union or organization. Legal definitions of each type of communication vary among states.

Fifth, the federal-level data only reflected campaign contributions to outside spending groups registered with the Federal Election Commission; this excluded contributions to outside spending groups that report to the Internal Revenue Service (eg, so-called 527 organizations, which can engage in electioneering communications). The federal-level data on campaign contributions also excluded direct advocacy communications paid for by corporations out of their own treasuries, which became legal following the ruling in the 2010 Supreme Court case of *Citizens United v Federal Election Commission*^26^; direct advocacy communications are referred to as independent expenditures in federal campaign finance regulations.

## Conclusions

From 1999 to 2018, the pharmaceutical and health product industry spent large sums of money on lobbying and campaign contributions to influence legislative and election outcomes. Understanding the spending of the pharmaceutical and health product industry on lobbying and campaign contributions can inform discussions about how to temper the influence of industry on US health policy.

## Notes

Supplement. **eTable.** Top 20 Recipients of Campaign Contributions From the Pharmaceutical and Health Product Industry in Presidential Elections, 1999 to 2018
