# Supplementary material for: Lobbying Expenditures and Campaign Contributions by the Pharmaceutical and Health Product Industry in the United States, 1999-2018
Source: JAMA Intern Med. 2020 Mar 3;180(5):1–10. doi: 10.1001/jamainternmed.2020.0146 (PMC7054854; doi:10.1001/jamainternmed.2020.0146)
Supplement: Supplement. — eTable. Top 20 Recipients of Campaign Contributions From the Pharmaceutical and Health Product Industry in Presidential Elections, 1999 to 2018 [file jamainternmed-180-688-s001.pdf]

Supplementary Online Content

Wouters OJ. Lobbying expenditures and campaign contributions by the pharmaceutical and health product industry in the United States, 1999-2018. *JAMA Intern Med*. Published online March 3, 2020. doi:10.1001/jamainternmed.2020.0146

eTable. Top 20 Recipients of Campaign Contributions From the Pharmaceutical and Health Product Industry in Presidential Elections, 1999 to 2018

This supplementary material has been provided by the authors to give readers additional information about their work.

© 2020 Wouters OJ. *JAMA Internal Medicine.*

eTable. Top 20 Recipients of Campaign Contributions From the Pharmaceutical and Health Product Industry in Presidential Elections, 1999 to 2018

Rank	Candidate (party)	Contributions received (millions, $)[a]	Contributions received (millions, $) by election[b]
1	Obama, Barack (D)	5.5	3.0 (2008) | 2.5 (2012)
2	Clinton, Hillary (D)	3.7	0.9 (2008) | 2.8 (2016)
3	Romney, Mitt (R)	3.0	0.5 (2008) | 2.5 (2012)
4	Bush, George W. (R)	2.4	0.8 (2000) | 1.6 (2004)
5	McCain, John (R)	1.0	0.0 (2000) | 1.0 (2008)
6	Kerry, John (D)	1.0	1.0 (2004)
7	Trump, Donald (R)	0.5	0.4 (2016) | 0.1 (2020)[c]
8	Giuliani, Rudolph W. (R)	0.3	0.3 (2008)
9	Bradley, Bill (D)	0.3	0.3 (2000)
10	Bush, Jeb (R)	0.2	0.2 (2016)
11	Paul, Ron (R)	0.2	0.1 (2008) | 0.1 (2012)
12	Gore, Al (D)	0.2	0.2 (2000)
13	Dean, Howard (D)	0.2	0.2 (2004)
14	Kasich, John (R)	0.1	0.03 (2000) | 0.1 (2016)
15	Dodd, Chris (D)	0.1	0.1 (2008)
16	Christie, Chris (R)	0.1	0.1 (2016)
17	Perry, Rick (R)	0.1	0.1 (2012) | 0.0 (2016)
18	Edwards, John (D)	0.1	0.05 (2004) | 0.05 (2008)
19	Carson, Ben (R)	0.1	0.1 (2016)
20	Gingrich, Newt (R)	0.1	0.1 (2012)
Total[d]	NA	19.3	NA

Abbreviations: D, Democrat; NA, not applicable; R, Republican.

[a] Data obtained from the Center for Responsive Politics. All monetary amounts were inflation-adjusted to 2018 dollars using the US Consumer Price Index.

[b] Reflected amounts received in individual election campaigns.

[c] Reflected contributions up until December 31, 2018.

[d] Numbers may not sum up to the total because of rounding.

© 2020 Wouters OJ. *JAMA Internal Medicine.*
